# Mental Health Nurses’ Experiences of Self-Care in Daily Practice: A Qualitative Study

**DOI:** 10.1177/00469580251375909

**Published:** 2025-10-07

**Authors:** Lise Sæstad Beyene, Christine Hammershaug, Linda Horne Mæland

**Affiliations:** 1 University of Stavanger, Norway; 2 Lovisenberg Diaconal University College, Oslo, Norway

**Keywords:** focus group interview, mental healthcare, mature care, nursing, self-care

## Abstract

In mental health nursing, nurses utilise their own personality and skills to create and maintain therapeutic relationships with patients. In such relationships, they come close to patients, show empathy, and hold patients’ emotional distress. Nursing in mental healthcare involves many emotionally challenging situations and is strenuous work. These demands can lead to high stress levels and feelings of inadequacy, which may negatively impact nurses’ quality of life and job satisfaction. Self-care is essential for mental health nurses to manage stress and maintain their well-being. Despite its importance, there is only limited research on nurses’ self-care. This study aims to explore mental health nurses’ experiences with self-care in their daily work. A descriptive phenomenological design was employed, and thematic analysis was conducted on data gathered from 1 focus group interview involving 4 mental health nurses working in acute mental health wards at 4 hospitals in central Eastern Norway. The theme *maturing into self-care is a process*, was based on 2 sub-themes: *becoming aware of self* and *learning to set boundaries for self.* The theme *dealing with self-care is challenging*, was based on 3 sub-themes: *distinguishing between work and leisure*, *balancing between own needs and those of others*, and *venting emotional pressure.* Mental health nursing is emotionally demanding, affecting mental health nurses’ quality of care and their own well-being. To sustain their ability to support others, formal structures must be in place to ease emotional burdens over time, creating a work environment where self-care is essential.

## Introduction

In mental health nursing, nurses utilise their own personality and skills to create and maintain therapeutic relationships with patients. In these relationships, mental health nurses (MHNs) develop close connections with patients, show empathy, and support them through emotional distress.^1^ MHN’s work implies therapeutic relational work, which is fundamental to all mental health treatment and associated with positive therapeutic outcomes for patients with mental health challenges.^2^ Therapeutic relational work requires MHNs ‘having a genuine interest in understanding the patient, engaging in reciprocal interaction with the patient, and meeting the patient so that they feel acknowledged’ as well as it requires them ‘having the ability to self-reflect and self-regulate’.^3(p17)^ This means that MHNs are to provide compassionate care for the patients, in addition to being aware of themselves. The intensity of their work can lead to high stress levels and feelings of inadequacy, which may negatively impact nurses’ quality of life and job satisfaction,^4-6^ as well as their sickness absence^7^ and retention in practice.^8,9^ Thus, essential for MHN’s ability to provide compassionate, high-quality therapeutic care for patients, and for their own well-being and job satisfaction is their self-care.^6,10-12^

Self-care can be described as strategies to establish and maintain own health and well-being.^13,14^ Strategies for self-care refer to practices, habits, and lifestyle choices that help to cope and manage negative stress.^13,15^ Self-care has to do with nurses identifying and coping with stress as well as enhancing their awareness of the importance of stress reduction.^16-18^ It relates to several life domains, including the physical, professional, relational, emotional, psychological, and spiritual.^13^ Nevertheless, in different practice contexts, nurses struggle with self-compassion and with allowing themselves to engage in self-care.^19,20^ MHNs report having a workday in which they are unable to attend to their basic physical needs, such as taking breaks, eating, drinking, and going to the toilet.^6,20^ Strategies for self-care include MHNs addressing workplace stressors and shaping a sense of cohesion so that one’s working life is viewed as comprehensible, meaningful, and manageable, thereby mitigating the risk of burnout.^21^ Despite the assumption that nurses who engage in forms of self-care are more likely to have greater job satisfaction,^10,22^ research on nurses’ self-care is sparse.^11^ This underscores the importance of looking closer into MHNs’ experience with self-care. This study aimed to explore MHNs’ experiences with self-care in their everyday working lives.

## Theoretical Perspective

A relevant perspective to understand MHN’s self-care is the theory of mature care, as described by the American psychologist Carol Gilligan.^23^ Mature care takes account of the interests of both the carer and the cared-for within a reciprocal relationship.^23,24^ While nurses are primarily responsible for addressing patients’ needs,^25^ they must also prioritise their own well-being to maintain their ability to provide effective care.^24^ In principle, a mature carer ‘should have as much care for oneself as for others’ ^24(p376)^ and balances between the needs of the self and those of others.^26^ Being a mature carer means reflecting on how to interact with others, how to understand and respond to patients, and how to care for and pay attention to oneself.^24^ Nurses who balance between the needs of their self and those of others are described as professionally mature, which is found to improve the well-being of both nurses and patients.^27^ As mature carers, nurses are better equipped to identify and respond to patients’ emotional distress, develop therapeutic relationships, and enhance their communication with patients.^26^ Furthermore, a mature career is setting clear boundaries and standing up for oneself, which implies ‘self-respect, and not being invaded by the feelings, opinions, and standards of other people’.^28(p130)^

## Methods

### Design

To address the research question, ‘How do MHNs experience self-care in their daily work?’ this study employed a descriptive phenomenological design.^29^

### Recruitment and Sample

This study was conducted in specialised mental healthcare in urban areas in Norway. The sample was strategic.^29^ To ensure that the participants had sufficient expertise and practical knowledge, the inclusion criterion required them to be registered nurses with more than 2 years of experience practising care for patients with mental disorders. The inclusion criteria ensured that all participants had substantial experience in mental care through their professional roles. There were no exclusion criteria. To recruit participants, snowball sampling was employed. Snowball sampling allowed the researchers to leverage professional networks to identify eligible participants who met the inclusion criteria, thereby enhancing the relevance and depth of the data.^29^ The second author contacted persons within her professional network and asked them to invite their colleagues and report contact information for those interested in receiving more information and participating. Seven registered nurses satisfied the inclusion criterion and were invited to participate and provided with an information letter. Three of them withdrew due to illness and workload.

The sample consisted of 4 registered nurses: 2 women and 2 men between 28 and 39 years of age. One had a master’s degree in mental health work and 1 had a post-graduate degree in mental health work. The participants had between 2 and 15 years of clinical experience in specialist mental health services. The participants worked in acute mental health wards in 4 different hospitals in central parts of Eastern Norway. Two of the participants were known to each other but had never worked together. They were unknown to the researchers.

### Data Collection

A focus group interview^30^ was conducted in November 2021, with the second author, who was a MHN and a master’s student, serving as the moderator. A semi-structured interview guide was used, featuring open-ended questions and key themes to steer the discussion, while allowing participants the flexibility to share their thoughts freely and introduce new perspectives.^29^ The participants were asked about their initial associations and personal experiences of self-care at work, and their thoughts on what enables nurses to prioritise self-care in their daily routines. They reflected on their attitudes and perceptions of self-care and were invited to share any additional thoughts or topics they felt should have been addressed. The interview was held in a neutral room at the nearby University. This location was chosen because it offered a neutral and comfortable atmosphere, allowing the participants to speak freely without the influence of public settings or workplace associations. The participants shared and reflected on their experiences of self-care in their everyday working lives as MHNs, as well as necessary conditions for prioritising self-care. The interview was audio recorded and lasted 100 min. After transcription, the material consisted of 25 pages of unprocessed text.

While 4 participants may seem limited, qualitative research prioritises depth over breadth. In this study, data saturation was assessed not by the number of participants alone, but by the richness and redundancy of the data collected. The interview was in-depth and yielded detailed insights. The participants were purposefully selected to represent key perspectives relevant to the research question, which further supports the adequacy of the sample size for this specific context. Given the wide range of experience of the 4 participants and the dynamic interaction within the group, comprising rich associations and narratives, it is considered that this focus group generated high information power.^31,32^

### Data Analysis

Thematic analysis was used to identify, describe, and analyse the data.^33^ The analysis proceeded through 6 phases and moved back and forth between the different phases. Firstly, the second author became familiar with the data by transcribing the audio recording into text and reading it carefully several times. The initial coding was conducted by way of identifying the codes and sorting them into groups. All possible codes were extracted from the full dataset to provide a comprehensive overview of patterns. Empirical patterns were then identified by way of searching for and comparing the code groups and labelling them with initial themes. The themes were then revised, merged, and reorganised by level of abstraction and then named by all authors to reflect their essence. Finally, the analysis of the data material based on the thematic map was written up in full text, ensuring consistency between the presented data and the study’s findings.^33^ The initial analysis was conducted in spring 2022, followed by a period of refinement, clarification, and finalisation during spring 2025 to enhance the quality and precision of the findings. An audit trail was maintained throughout the analysis process to ensure transparency and accountability. All data handling steps, methodological decisions, and analytical procedures were documented systematically, allowing for traceability and reproducibility of the results.^29^

Ensuring high quality, important methodological aspects regarding this study are reported in the COREQ checklist.^34^

### Ethical Considerations

This study followed the research ethics principles outlined by the World Medical Association.^35^ Key ethical considerations in the focus group methodology included ensuring confidentiality, obtaining informed consent, and maintaining trust. Personal data collected during the study was anonymised and encrypted to protect the participants’ privacy. Participants were guaranteed anonymity and confidentiality, and the informed consent process highlighted their voluntary participation and their right to withdraw at any time. Clear communication and respect for participants’ time and vulnerability were prioritised throughout.

Sharing one’s experience with self-care may entail to sense of vulnerability. Conducting a focus group interview made it possible to create a sense of trust and security between the researcher and the participants before, during, and after the interview.^29^ The project was approved by the Norwegian Social Science Data Service.

## Results

The thematic analysis illuminated 2 themes related to the participants’ experiences of self-care in their everyday working life in mental healthcare: *maturing into self-care is a process* and *dealing with self-care is challenging.* Table 1 provides an overview of the themes and subthemes.

**Table 1.** Overview of Themes and Subthemes.

Themes	Maturing into self-care is a process	Dealing with self-care is challenging
Subthemes	Becoming aware of self Learning to set boundaries for self	Distinguishing between work and leisure Balancing between own needs and those of others Venting emotional pressure

### Theme 1. Maturing Into Self-Care is a Process

Based on the participants’ descriptions, we can perceive a development from their early days as new graduates, when they often said yes to everything and were unaware of their own needs, to a gradual realisation of the importance of setting boundaries and taking care of themselves. The participants stated that their education did not adequately prepare them for the realities of the job, such as the importance of self-care. The lack of focus on self-care in nursing education and the unclear delineation of the nursing role were associated with factors related to a limited focus on self-care. In retrospect, the participants could see that, as novices, they were not in touch with their own needs and agreed to significant extra workloads. They shared that they gradually became aware of the importance of self-care as they felt the consequences of the workloads. Maturing into self-care was described as a process where the participants became more aware of themselves through everyday work experiences that made them understand that self-care is necessary in order to ‘endure’, and in this way they learnt to set boundaries for themselves. The participants gradually recognised that to provide quality care to mentally ill patients, they first needed to take care of themselves.

*Becoming aware of self:* When looking back on their time as newly graduated nurses, the participants recalled lacking self-awareness regarding their own needs and limits. They were focussed primarily on meeting the demands of their job and the expectations of their colleagues and leaders, and they were not initially aware of themselves and what they needed for themselves. This lack of self-awareness led them to take on excessive workloads and responsibilities, often at the expense of their own well-being. The participants had internalised beliefs about needing to be self-sacrificing and always put others first. One nurse shared the following:

*It feels like there is more focus on the patient’s well-being than the nurse’s. (. . .) When something happens, the focus is always on the patient first, because it’s about the patient’s life or health*. (Nurse 2)

The participants felt that they should neglect their own feelings and deny what they needed for themselves. However, they noticed their own physical and emotional signs of strain, such as fatigue, irritability, and a sense of being overwhelmed, a feeling of becoming a person who they did not want to be:

*What strikes me is that you put so much empathy and love into your work, and then when you get home, you have nothing left to give. And it affects your free time and personal life so much that it no longer feels worthwhile.* (Nurse 2)

The participants became aware that they ran out of empathy and felt flattened when they met with patients. Such experiences served as wake-up calls, prompting them to reflect on themselves and what they needed. With the support of colleagues and mentors, who encouraged a more balanced approach to caregiving, as well as reflection on their own experience in mental healthcare, the participants became more attuned to their emotional responses to work-related stressors and learnt to acknowledge and address these feelings rather than ignore them. In this way they became aware of themselves and matured into self-care. By becoming more self-aware, participants were able to better understand their own needs and limits, which led them to engage in more constructive self-care practices, such as taking the time to think about their experiences, journalling, and discussing their feelings with trusted colleagues or mentors. In their experience, engaging in reflective practices was important for them to be able to identify patterns in their behaviour towards patients and also influenced their own well-being. Self-awareness was therefore a crucial component of their journey towards maturing into self-care.

*Learning to set boundaries for self:* As new graduates, the participants often agreed to all tasks and responsibilities, driven by a fear of making mistakes and a desire to please colleagues and leaders. Over time, the participants realised the negative impact on their physical and mental health of not setting boundaries around what they would engage in. They started to understand that saying ‘yes’ to everything was unsustainable and detrimental to their own well-being and they emphasised the importance of being assertive and communicating their boundaries to their leader and colleagues:

*I didn’t think much about it before; it was only later that I started thinking about self-care as setting boundaries for myself. In the beginning, you just went all in with double shifts and everything. And eventually, you realise that if you keep going like that, it will become really heavy in the long run. Through experience, I understand this now.* (Nurse 4)

Gradually, the participants learnt to say ‘no’ to additional tasks and responsibilities that they felt were beyond their capacity or beyond their job scope. Learning to set boundaries for themselves helped them to managing stress better, which improved their own well-being and allowed them to provide better care to patients. They experienced the process of maturing into self-care as crucial for their long-term sustainability in mental health nursing.

### Theme 2. Dealing With Self-Care Is Challenging

Dealing with self-care involved the participants trying to figure out how to prioritise and take care of themselves in their daily practice. The participants found self-care to be challenging in daily nursing practice. They described a working culture that did not always support their expression of their own needs or vulnerability. They indicated that it could be difficult to show their colleagues and leaders that they were affected by patients’ stories, and they felt that expressing their own emotions was often seen as a weakness. The participants pointed out that in the absence of their leaders’ lack of support and acceptance, it was difficult for them to deal with the tough impressions from their everyday work. The emotional demands of mental health nursing were found to be overwhelming, which prompted them to reevaluate their career choices. Some of the participants shared having thought about leaving their jobs if there were no opportunities to prioritise self-care at work. They realised that prioritising their well-being by leaving their demanding job and working in a less demanding setting might be necessary for them to deliver high-quality patient care. Dealing with self-care was a matter of the participants distinguishing between work and leisure, balancing between their own needs and those of others, and venting emotional pressure.

*Distinguishing between work and leisure:* The participants discussed the importance of clearly distinguishing between work and leisure. One way of dealing with this was to choose a point on their way home when they could stop thinking about work. However, they found this to be challenging. They conveyed that they struggled to leave work-related thoughts and emotions behind when they stepped out of the workplace. At work, they dealt with patients’ suffering, trauma, and emotional distress – an emotional burden that was difficult to put away when the shift was over. The participants indicated that strong impressions cannot be erased from their minds when they leave work but remain as memories and mark their leisure time. They could find themselves grappling with feelings of anger, sadness, fear, and frustration that lingered long after they had left the workplace. As one of the participants stated:

*The problem with acute psychiatry is that it becomes a kind of window into all that is dark and horrible in the world. It’s pretty grotesque, but if you don’t look into that window, you’re really not doing your job. You have to listen to someone recounting how she was exploited in a paedophilic poker game during her childhood, right? And those kinds of stories stay with you, lingering in your mind as you return to your own life.* (Nurse 3)

The participants referred to the importance of having hobbies and interests outside of work to help them disconnect and recharge. Having something they felt passionate about that they can engage in during leisure time is good self-care. For coping with emotional distress, some of the participants found that physical activities like running were ideal. However, they recognised that this is not sustainable over the long term. While physical activities temporarily alleviate stress and can be used as strategies for self-care, they do not fully address the emotional impact of patient encounters.

*I listened to music. I said to a colleague, ‘Can you watch my patients while I step out for 15 minutes?’, without saying what I was going to do. Just to get a little breather and think about something else. For me, it worked at first.* (Nurse 2)

The participants highlighted the need for formal support at work, which they felt was lacking, to help alleviate emotional pressure over time.

*Balancing between own needs and those of others:* The participants found that their own needs were often at odds with the needs of their patients, colleagues, or leader. This required them to find a balance between their own needs and those of others. The participants accomplished this by trying to speak up and saying ‘no’ when the leader wanted to impose new or additional tasks on them. For instance, they could insist on taking a 30-min lunch and ask colleagues for assistance so that they could withdraw from the ward for a breather. The participants said that they would occasionally ask to switch patients or work in teams to better manage their workload and stress levels. They also tried to be proactive about communicating with their leader if they noticed that the duty team scheduled for the next day was understaffed, thus ensuring that there were enough team members to handle the workload effectively. They found that this helped them to achieve a more balanced and supportive work environment. They did point out, however, that it was difficult to present and express what they needed, but they found that if several colleagues jointly expressed what they needed for themselves, a climate was created that balanced the needs of the nurses and the patients. One participant stated:

*After a period of a lot of violence at the ward. . . We told the leader, ‘We can’t go on like this any longer, we have to do something different’ and this improved the situation. We became more generous with each other and perhaps more open, said things as they were, and people understood. It was pretty cool to be part of that change. . .you were seen as tough if you managed to speak up.* (Nurse 4)

The participants felt a sense of professional responsibility and a strong sense of duty towards the needs of their patients, colleagues, and leader. The tension between various expectations became clear in situations where they had to decide whether to take on an extra shift to ensure staffing or time off to prioritise their own need for rest and recovery. The participants found that leaders expected them to be available and responsive, often beyond their regular working hours. They felt pressured to meet these expectations in order to avoid disappointing their leaders or letting their colleagues down. They also reported that their colleagues expected them to help out with additional tasks, for instance, which made them struggle with feeling guilt for saying no to these colleagues, who also were under pressure, which added to their own stress:

*When you try to say no (to double shifts), it also becomes exhausting. I was kind of completely drained afterward because I kept thinking about how they needed me to work double shifts and overtime every single week, and it’s like, ‘We don’t have anyone for the night shift tonight, so who can work?’ It almost feels worse to pull back and say no than to just go to work.* (Nurse 2)

The participants felt that admitting that they needed a break or expressing emotional distress could be seen as a sign of weakness, which made them feel that they needed to hide their struggles and push through their challenges without seeking help. Feelings of guilt and a strong sense of responsibility towards patients and colleagues made it difficult for the participants to prioritise self-care. They struggled with the fear that prioritising self-care would negatively influence patient care or burden their colleagues:

*Yeah, you have to be so tough all the time. It’s a bit like, ‘You’ve chosen to work here, so you just have to handle it,’ and one tries to be tougher than the other. If someone breaks down, colleagues and even the leader can start to think that the person isn’t suited for this job.* (Nurse 1)

*Venting emotional pressure:* The participants said that their work as MHNs exposed them to intense and often distressing experiences, which created a buildup of emotional pressure that needed to be released for them to maintain their own mental well-being. They discussed the importance of finding ways to ‘vent’ or release this pressure to prevent it from negatively affecting them and their ability to care for patients. The participants sought support from colleagues and shared their feelings and experiences with colleagues who understood the nature of their work. One participant said:

*I have worked in places with diverse cultures. I felt better when we could talk with colleagues about the things we experienced, and we would sit down together to release some pressure. You must let it out somewhere, the frustration. I think it’s all about the colleagues and opportunities to vent out with them.* (Nurse 4)

For the participants, venting their experiences was a process that reduced stress and prevented the buildup of negative emotions. They emphasised the need for a supportive work environment among colleagues that encourages open communication and emotional expression, and they suggested that regular debriefing sessions, peer support groups, and access to professional supervision could help them to vent emotional pressure more efficiently. However, some participants experienced that if they were already feeling emotionally overloaded, they could feel overwhelmed if their colleagues needed to tell them about challenging experiences and vent their feelings. They felt stretched between the responsibility of supporting a colleague and maintaining protective boundaries to manage their own needs.

When trying not to burden their colleagues by not sharing their own emotional pressure, the participants felt left alone with their impressions in their everyday work, with no occasion to talk openly about their experiences with patients. The participants said they felt isolated in dealing with the emotional weight of their work, not just the everyday impressions but also stronger impressions of a more violent and potentially stressful nature. While recognising the potential of talking about their experiences, they acknowledged the difficulty of finding someone who truly understood the depth of their emotions. In a challenging work environment, they found themselves carrying these impressions by themselves. They discussed not having anyone to talk to about what they were experiencing in their everyday working lives, whether at work or privately. As one of the participants stated:

*You don’t talk about what’s difficult, because who can you talk to? At work, you have people who are just supposed to be so tough and handle everything, and at home, you have a partner who doesn’t understand anything about what you’re doing or what you’re going through at work. So you end up sitting alone with it.* (Nurse 3)

The participants discussed lacking formal support, such as debriefing sessions and professional supervision, to help them process their emotions. This lack of support and the feeling of being left alone were considered a challenge to self-care. All the participants expressed expecting and needing a leader who fosters a culture that values and supports self-care and emotional well-being, as well as formal and distinctive support mechanisms at work.

## Discussion

This study aimed to explore MHNs’ experiences with self-care in their everyday working lives and reveal experiences of *maturing into self-care is a process* and *dealing with self-care is challenging.*

This study’s results indicate that self-care for MHNs begins with the recognition of its necessity. This highlights the relevance of integrating self-care into nursing education, which can be seen as a proactive approach to teaching nurses about self-care, setting boundaries, and reflecting on their values early on.^15^ This can help MHNs manage the emotional and practical demands of their roles, potentially reducing role conflict and the negative health outcomes of neglecting self-care.^6^ Furthermore, training nurses in self-care may increase their awareness of self in a caring relationship^36^ and affect the quality of patient care.^37^

Research shows that the disparity between MHNs’ needs and those of patients, leaders, and colleagues can lead to role conflict, potentially leading to negative health outcomes and extended sick leave.^7^ Therefore, it is important to prioritise self-care early, particularly during nursing education.^16,37^ However, this study found that MHNs may not fully grasp their own needs or limitations until they gain significant caregiving experience, especially within specialist mental health services. While knowledge of self-care in nursing education may foster greater self-compassion and a sense of responsibility for well-being, it remains uncertain whether MHNs would practice self-care, as implementation challenges may persist if resources remain limited.^11^

This study shows that maturing into self-care is emotionally challenging and can lead to feelings of guilt and stress for MHNs, as it conflicts with what is expected of them as caregivers. The emotional cost of prioritising self-care highlights the moral demands on the nursing profession, which can undermine nurses’ integrity over time.^21^ MHNs can more easily take care of their own needs if they are working in line with their own values, setting boundaries, and saying ‘no’ when work violates their values.^7^ This requires them to cultivate a conscious awareness of their values and maintain a reflexive attitude towards themselves as nurses.

This study indicates that MHNs frequently set their personal needs aside in certain areas of the job, particularly patient interactions. It is expected of them,^25^ and they are often admired for their self-sacrifice.^24^ However, it can be questioned whether they do so out of necessity in patient encounters or because they have been trained under the traditional altruistic care ideal. The altruistic care ideal concerns nurses sacrificing their own needs for the benefit of the patient’s well-being.^38^ It may seem clear that helping the sick is the right thing for a nurse to do, while prioritising oneself is wrong when others are worse off. On the other hand, the altruistic care ideal may hinder MHNs from prioritising what they need for themselves and from attaining a reciprocal relationship as a mature carer.^24^ Self-care is often perceived as negative or selfish, leading nurses to hide their self-care efforts at work due to role expectations.^11^ The results of this study align with Pettersen^24^ and confirm that in a culture where satisfying others’ needs is highly valued, self-care is undervalued. This likely leads to uncertainty about professional responsibilities and challenges in terms of setting personal boundaries. Therefore, navigating the tension between various expectations is more than a matter of working long enough to gain security, self-confidence, and the ability to voice one’s own needs and boundaries. It also involves understanding oneself, including one’s characteristics, unique needs, and tolerance limits within the nursing role and the work environment.^39^ In the context of other research, it appears that setting aside personal needs in patient encounters can lead to role conflict and tension for nurses.^7^ Several theories suggest that it should be possible for nurses to maintain self-awareness and focus on their personal boundaries while being present with patients, and that this would strengthen rather than diminish the quality of care.^13,23,24^ Perhaps, then, it should never be necessary for MHNs to set aside either their self-awareness or their focus on their own needs in their everyday work.

The results of this study confirm that MHNs can be emotionally affected by the impressions they receive in their relational work, generating significant pressure that requires processing. Discussion of their emotional burdens helps them to relieve internal pressure and is perceived as a form of self-care in their daily work. However, they find it challenging to share and vent their emotional pressure. If MHNs expect colleagues to listen to them, there is the reciprocal expectation that they should listen to colleagues. This can create a strenuous workday permeated by emotional impressions and pressure. It has been found that even MHNs with significant work experience and awareness of setting boundaries between their own needs and those of others often struggle to let colleagues know when they lack the capacity to listen to their experiences. This requires them to regulate their emotions not only in interactions with patients but also in meetings with colleagues; if they do not, it could negatively affect their emotional well-being and sickness absence.^7^

It is appropriate to question who is responsible for MHNs’ self-care at work, as it may be a challenge for them to manage this on their own. Leaders must assume responsibility for addressing MHNs’ needs by facilitating methods tailored to the workplace and daily work routines.^40^ Methods for clinical supervision that include group reflection, venting, defusing, and debriefing are shown to be health-promoting and to support MHNs.^41-43^ Processing impressions by talking about them is associated with self-care, but for venting to be health-promoting rather than a burden on the environment, the responsibility must lie with the employer rather than the individual employees. Ideally, MHNs should have time and space to reflect on patient interactions, supported by routine professional guidance for structured team processing.^43^ Given the crucial role that MHNs play in delivering care, leaders must commit to supporting their MHNs’ well-being.^21^ If MHNs are left to handle their emotional pressure on their own, it creates an extra layer of stress, and adopting a proactive approach to self-care is important.^15^ This highlights the need for formal spaces for venting, reflecting, and processing.^43^

### Methodological Strengths and Limitations

The diverse and unique self-care experiences of the participants, combined with the interactive nature of the focus groups, encouraged the participants to engage with one another. This interaction fostered new insights and highlighted shared experiences, resulting in rich and comprehensive data material. The thematic analysis of this data material has provided valuable insights into the participants’ experiences and perspectives, offering an in-depth understanding of their views. Despite the small sample size, the data collection and thematic analysis have captured central aspects of MHNs’ experiences of self-care. The participants’ reflections were consistent across different departments, thus supporting the study’s credibility. However, as the participants represent only a small sample, the findings cannot be generalised to a broader population, and different results might have been obtained had participants been recruited from other nursing contexts or with a larger sample.

The reliance on the researchers’ interpretations in the thematic analysis has introduced a level of subjectivity and potential for bias. To safeguard trustworthiness, the authors have cooperated in reflecting on the results and performing their interpretations in alignment with the participants’ original meaning. While member checking can enhance credibility, it was not conducted in this study due to practical constraints. Instead, credibility was supported through rigorous analytical procedures, including systematic coding, peer debriefing, and reflexive journaling. These strategies helped ensure that the findings accurately reflect participants’ perspectives.^29^

Despite these limitations, the study’s methodological strengths allow for a comprehensive understanding of the MHNs’ experiences and thus contribute valuable knowledge to the field.

## Conclusion and Implications

This study has found that maturing into self-care is a process that involves becoming aware of self through experience and reflections and learning to set boundaries for self through practical experiences and realisations. Dealing with self-care is challenging. Various activities that help MHNs distinguish between work and leisure are used as strategies for self-care, although these seem to only temporarily alleviate emotional stress. Balancing between own needs and those of others and venting emotional *pressure* seem to be too challenging for MHNs to handle on their own.

There is a need for formal support in MHNs’ everyday work to facilitate and alleviate emotional pressure over time, ensuring that MHNs have a space for self-care. Leaders should ensure that MHNs prioritise their self-care and well-being by facilitating formal clinical supervision or other interventions to help them vent emotional pressure. Self-care among MHNs should be encouraged by leaders to strengthen a supportive and healthy workplace culture. It is important to incorporate self-care into nursing education to help MHNs mature into self-care practices.

Future research should investigate whether MHNs who take care of themselves enjoy their job more and thus remain in it longer.

## Supplemental Material

sj-docx-1-inq-10.1177_00469580251375909 – Supplemental material for Mental Health Nurses’ Experiences of Self-Care in Daily Practice: A Qualitative Study Supplemental material, sj-docx-1-inq-10.1177_00469580251375909 for Mental Health Nurses’ Experiences of Self-Care in Daily Practice: A Qualitative Study by Lise Sæstad Beyene, Christine Hammershaug and Linda Horne Mæland in INQUIRY: The Journal of Health Care Organization, Provision, and Financing
