# Supplementary material for: Mental Health Nurses’ Experiences of Self-Care in Daily Practice: A Qualitative Study
Source: Inquiry. 2025 Oct 7;62:00469580251375909. doi: 10.1177/00469580251375909 (PMC12504838; doi:10.1177/00469580251375909)
Supplement: sj-docx-1-inq-10.1177_00469580251375909 – Supplemental material for Mental Health Nurses’ Experiences of Self-Care in Daily Practice: A Qualitative Study [file sj-docx-1-inq-10.1177_00469580251375909.docx]

# COREQ (COnsolidated criteria for REporting Qualitative research) Checklist

| Topic | Item No. | Guide Question/Description | Reported on  Page No. |
| --- | --- | --- | --- |
| Domain 1: Research team and reflexivity | | | |
| Personal characteristics | | | |
| Interviewer/facilitator: | 1 | Who conducted the interview or focus group? | 5 |
| Credentials: | 2 | What were the researcher's credentials? e.g. PhD, MD | Title page |
| Occupation: | 3 | What was their occupation at the time of the study? | 5 |
| Gender: | 4 | Was the researcher male or female? | N/A |
| Experience and training: | 5 | What experience or training did the researcher have? | Title page |
| Relationship with participants | | | |
| Relationship established: | 6 | Was a relationship established prior to study commencement? | 5 |
| Participant knowledge of the interviewer: | 7 | What did the participants know about the researcher? | 5 |
| Interviewer characteristics: | 8 | What characteristics were reported about the interviewer/facilitator? | N/A |
| Domain 2: Study design | | | |
| Theoretical framework | | | |
| Methodological orientation and theory: | 9 | What methodological orientation was stated to underpin the study? | 4 |
| Participant selection | | | |
| Sampling: | 10 | How were participants selected? | 4 |
| Method of approach: | 11 | How were participants approached? | 4 |
| Sample size: | 12 | How many participants were in the study? | 5 |
| Non-participation: | 13 | How many people refused to participate or dropped out? | 4 |
| Setting | | | |
| Setting of data collection: | 14 | Where was the data collected? | 5 |
| Presence of non-participants: | 15 | Was anyone else present besides the participants and researchers? | 5 |
| Description of sample: | 16 | What are the important characteristics of the sample? | 5 |
| Data collection | | | |
| Interview guide: | 17 | Were questions, prompts, guides provided by the authors? | 5 |
| Repeat interviews: | 18 | Were repeat interviews carried out? | 19 |
| Audio/visual recording: | 19 | Did the research use audio or visual recording? | 5 |
| Field notes: | 20 | Were field notes made during and/or after the interview or focus group? | N/A |
| Duration: | 21 | What was the duration of the interviews or focus groups? | 5 |
| Data saturation: | 22 | Was data saturation discussed? | 5-6 |
| Transcripts returned: | 23 | Were transcripts returned to participants for comment and/or correction? | 19 |
| Domain 3: Analysis and findings | | | |
| Data analysis | | | |
| Number of data coders: | 24 | How many data coders were involved in the analysis? | 6 |
| Description of the coding tree: | 25 | Did authors provide a description of the coding tree? | 7 |
| Derivation of themes: | 26 | Were themes identified in advance or derived from the data? | 6 |
| Software: | 27 | What software, if applicable, was used to manage the data? | N/A |
| Participant checking: | 28 | Did participants provide feedback on the findings? | 19 |
| Reporting | | | |
| Quotations presented: | 29 | Were participant quotations presented to illustrate the themes/findings? | 8-14 |
| Data and findings consistent: | 30 | Was there consistency between the data presented and the findings? | 6 |
| Clarity of major themes: | 31 | Were major themes clearly presented in the findings? | 7, 8, 10 |
| Clarity of minor themes: | 32 | Is there a description of diverse cases or discussion of minor themes? | 7-15 |

Developed from: Tong A, Sainsbury P, Craig J. Consolidated criteria for reporting qualitative research (COREQ): a 32-item checklist for interviews and focus groups. *International Journal for Quality in Health Care*. 2007. Volume 19, Number 6: pp. 349 – 357
